# Prevalence and severity of antipsychotic related constipation in patients with schizophrenia: a retrospective descriptive study

**DOI:** 10.1186/1471-230X-11-17

**Published:** 2011-03-08

**Authors:** Marc De Hert, Liesbeth Dockx, Chiara Bernagie, Bie Peuskens, Kim Sweers, Stefan Leucht, Jan Tack, Stefan Van de Straete, Martien Wampers, Joseph Peuskens

**Affiliations:** 1 University Psychiatric Centre Catholic University Louvain, Leuvensesteenweg 517, 3070 Kortenberg; 2 Medical faculty Campus Gasthuisberg Catholic University Louvain, O&N2, Herestraat 49 - bus 700, 3000 Leuven, Belgium; 3 Department of Psychiatry and Psychotherapy, Technische Universität München, Klinikum rechts der Isar, Ismaningerstr. 22, 81675 Munich, Germany; 4 Department Gastroenterology Catholic University Louvain, O&N I Herestraat 49 bus 701, 3000 Leuven, Belgium

## Abstract

**Background:**

Antipsychotic are the cornerstone in the treatment of schizophrenia. They also have a number of side-effects. Constipation is thought to be common, and a potential serious side-effect, which has received little attention in recent literature.

**Method:**

We performed a retrospective study in consecutively admitted patients, between 2007 and 2009 and treated with antipsychotic medication, linking different electronic patient data to evaluate the prevalence and severity of constipation in patients with schizophrenia under routine treatment conditions.

**Results:**

Over a period of 22 months 36.3% of patients (99) received at least once a pharmacological treatment for constipation. On average medication for constipation was prescribed for 273 days. Severe cases (N = 50), non-responsive to initial treatment, got a plain x-ray of the abdomen. In 68.4% fecal impaction was found.

**Conclusion:**

A high prevalence of constipation, often severe and needing medical interventions, was confirmed during the study period. Early detection, monitoring over treatment and early intervention of constipation could prevent serious consequences such as ileus.

## Background

Antipsychotics are the cornerstone of the treatment of schizophrenia and related psychotic disorders. There is a growing interest in the physical co-morbidities and somatic side-effects of these agents [1-6]. Constipation is a well-known but poorly researched side-effect of antipsychotics [7-9].

Recent reviews showed that constipation is prevalent and can lead to severe consequences and even premature mortality [7,10].

Talley et al. evaluated the association between medication and constipation [11]. Antipsychotic users had a 1.9 higher risk to develop constipation compared to non-users. Stanniland and Taylor reported rates of 1.5% to 25% compared to placebo [12]. In clozapine users constipation is among the top 4 reported side-effects with prevalence rates ranging from 14 up 60% [13-15]. In extreme cases this may lead to bowel obstruction and paralytic ileus which, if not detected and treated early may lead to premature mortality [14,16-18]. Palmer et al. described 102 cases of severe gastrointestinal side-effects on clozapine of which 28 were fatal [14]. Mortality on clozapine due to constipation could be higher than that of agranulocytosis [7].

At our University Hospital an pharmacy audit in 2008 showed that at least 1 out of 3 adult patients with schizophrenia were at least once treated for constipation during hospitalization. In 2009 the total expenditure for medication for constipation exceeded 40,000 USD per year. As a comparison this constituted 5 times the expenditure of anticholinergic drugs for motor side-effects, 30% of the cost of depot antipsychotics or 7% of the total cost of antipsychotics [2,10].

The aim of the current study was to evaluate the prevalence and severity of constipation in routine treatment in patients with schizophrenia.

## Methods

We performed a retrospective electronic record linking study (medical records, pharmacy data, somatic data and radiological data) on all admitted patients, both inpatients and patients in day-hospitalization, between June 2007 and March 2009, with a clinical DSM-IV diagnosis of schizophrenia treated with antipsychotic medication in a University Psychiatric Hospital, with a regional Catchment area of 300,000 inhabitants. Patients were only included if they were not treated with laxatives at first inclusion.

There were no other in- or exclusion criteria. The association between specific antipsychotics was not assessed because of the retrospective nature of the study and potential polypharmacy which could confound the results.

Constipation was defined as have at least one new prescription of a laxative.

Descriptive statistics were performed with statistical analysis software (SAS, Statistical Analysis System, Carey NC).

Patients had given informed consent for anonymous data analysis and the study was approved by both the local scientific and ethical committee.

## Results

Over the 22 months observation period there were 371 admissions of 273 individual patients with schizophrenia. The mean age of the sample was 40.1 years (±13.5, range 17 to 82) and 65.6% were male. The mean duration of admission (including day hospitalization) was 472 days (±1,073, median 99.3, range 0.02 to 6,291).

During the admissions there were 1,751 different prescriptions of antipsychotics. The largest groups were second generation antipsychotics (Table 1.). Over the study period there were no major trends of change of the relative distribution of individual drugs. There were 123 prescriptions of anticholinergic agents (89.4% biperidene).

**Table 1:** Prescription of antipsychotics (N prescriptions and %).

Antipsychotic	Product	Frequency	%
**2**^**nd **^**generation (Atypical)**	Aripiprazole (Abilify^**^®^**^)	202	11.5
	Clozapine (Leponex^®^)	165	9.4
	Risperidone (Risperdal^®^)	223	12.7
	Sertindole (Serdolect^®^)	43	2.5
	Quetiapine (Seroquel^®^)	126	7.2
	Amisulpride (Solian^®^)	89	5.1
	Olanzapine (Zyprexa^®^)	204	11.7
**1st generation (Typical)**	Low-potency	301	17.2
	High-potency	209	11.9
**Long-acting (Depot)**	Risperidone (Risperdal Consta**^®^)**	115	6.6
	Typical depot	73	4.2
			
**Total**		1,751	100

Ninety nine patients (36.3%) had at least 1 new pharmacological intervention for constipation. In total 11 different interventions were recorded (Table 2.). The most frequently used drugs were the macrogols 4,000 (30.6%) and 3,350 (22.5%) followed by the stimulant laxative sodiumpicosulphate (25.4%). These 3 constituted 75% of all drugs used to combat constipation.

**Table 2:** Pharmacological treatment of constipation (N prescriptions and %).

Product	2007		2008		2009		Total period	
	**Freq**	**%**	**Freq**	**%**	**Freq**	**%**	**Freq**	**%**

**Bisacodyl + enema (bowel preparation enema like)**	0	0	1	0.8	0	0	1	0.4
**Ispaghula**	1	1.4	1	0.8	0	0	2	0.7
**Lactulose**	2	2.7	8	6	2	3.1	12	4.4
**Macrogol (bowel preparation enema like)**	19	26	2	1.5	0	0	21	7.8
**Macrogol 3,350**	8	11	36	27	17	26.2	61	22.5
**Macrogol 4,000**	19	26	40	30	24	36.3	83	30.6
**Sodiumpicosulphate**	17	23.1	34	25.6	18	27.7	69	25.4
**Senna**	0	0	0	0	1	1.5	1	0.4
**Sodium phosphate (enema)**	5	6.9	7	5.3	2	3.1	14	5.2
**Soap enema**	0	0	2	1.5	0	0	2	0.7
**Water enema**	2	2.7	2	1.5	1	1.5	5	1.9
								
**Total**	73	100	133	100	65	100	271	100

Over the study period 271 different treatments periods were recorded in 99 patients. Of these 16% were treatments with enemas, for constipation episodes non-responsive to oral treatments. 68% of the constipation treatments lasted longer than 1 week. Only enemas were prescribed for shorter periods (mostly a single intervention). For lactulose and macrogol 4,000 90% of prescriptions lasted longer than a week. The mean duration of prescriptions, excluding enemas, was 273 days (±603, median 49, range 1 to 4,854) and was different for specific drugs (lowest for macrogol 4,000 195 days (±233, median 124, range 1 to 1,124), followed by sodiumpicosulphate 307 days (±716, median 73, range 1 to 3,766) and longest for macrogol 3,350 522 days (±922, median 38, range 1 to 3,766)).

There were 114 referrals (N patients 54) to the somatic department for additional treatment and follow-up of constipation. In 89.5% of cases this resulted in prescription of oral medication, 7.9% enemas and 8.8% other interventions such as advice on diet, hydratation and physical activity (multiple interventions possible).

Of the referred patients 92.6% (N = 50) were examined with a plain x-ray of the abdomen. A total of 99 of x-ray examinations of the abdomen were registered. Only in 5.1% (N = 5) were negative. In 26.5% (N = 26) fecal accumulation was observed, while fecal impaction was present in 68.4% (N = 68).

## Discussion

This is the first study systematically evaluating the prevalence and severity of constipation in patients with constipation under routine treatment conditions. Constipation was highly prevalent and often required pharmacological intervention (36.3% of patients).

Hiele reported a prevalence of constipation between 2 and 28% in the Belgian general population [19]. In a review on the prevalence of constipation in Europe showed an average prevalence of 17.1% [20]. Other population studies estimate signs and symptoms of chronic constipation in 10 to 20% of individuals of a general practice sample [11]. In all these studies the higher prevalence rates were found in older people.

In our study with a younger population of patients with schizophrenia constipation rates were higher than in general population samples [11,19,20]. The true prevalence in patient samples may be even higher since we identified constipation only in patients who were either referred to the somatic department or who received treatment for constipation.

Our study confirms the scarce literature on antipsychotic associated constipation in patients with schizophrenia. Additional risk factors in this patient group might be diet and lack of exercise related [18,21]. Anticholinergic medication can also induce gastrointestinal symptoms, including constipation [7]. Schizophrenia as such and its treatment with antipsychotics could be accompanied with a higher discomfort or pain threshold, which could be a reason why these patients report somatic complaints later or less frequently [22,23]. This could lead to late or under-diagnosis of constipation. Next to a higher pain threshold negative symptoms could also be associated with more indifference and problems with adequately expressions of pain sensations [22,23].

Constipation was not only frequent but also severe. This was confirmed by the long duration of constipation treatment, of more than 250 days on average. The use of enemas was not rare (16% of constipation treatments). Constipation can lead to fecal impaction, resulting in bowel obstruction and perforation [7]. Multiple cases with a fatal outcome have been reported as a result of perforation, asphyxiation of fecal vomitus and fecal impaction resulting in necrosis [18]. The severity of constipation associated with antipsychotic drug use was also confirmed in this study with the high rate of fecal impaction, a risk factor for more severe and potentially lethal complications [7]. A recent register-based study focusing on cases of ileus in patients with schizophrenia showed that both anticholinergic drugs and antipsychotics with anticholinergic properties were associated with fatal cases [24].

Given the potential severe consequences of constipation early detection, monitoring over the course of treatment and early treatment are important. Side-effects are an important reason for non-compliance and constipation is also associated with poorer health related quality of life [25]. Next to treatment with laxatives a reduction of antipsychotic dose has been proposed in the literature [26].

Limitations of this study are the retrospective design relying on electronically recorded data and not direct patient assessments. The study is mono-centric which can impact on generalizability. We did not evaluate the association between specific antipsychotic medication and the prevalence nor incidence of constipation. No data was available on constipation treatments prior to the studied period. A last limitation is that no data was collected on outpatients.

Future research should focus on predictors and the effectiveness of preventive measures and the different treatment options, as well as the underlying pharmacological mechanisms (e.g. predictors, differences between antipsychotic agents, timing of onset of constipation).

## Conclusions

Constipation associated with antipsychotic treatment is frequent in patients with schizophrenia. It can be severe when early detection fails. It leads to long-term prescription of pharmacological interventions. Clinical staff should be aware of this association and actively screen, monitor and provide early treatment. Preventive action with advice on diet, hydratation and adjustment of physical activity levels should be promoted.

## Competing interests

MDH has been a consultant for, received grant/research support and honoraria from, and been on the speakers/advisory boards of Astra Zeneca, Bristol-Myers Squibb, Eli Lilly, Janssen-Cilag, Lundbeck, Pfizer and Sanofi-Aventis.

SL received speaker/consultancy/advisory board honoraria from BMS, Eli Lilly, Essex Pharma, GlaxoSmithKline, Janssen/Johnson and Johnson, Lundbeck, Pfizer and Sanofi Aventis, Eli Lilly and Sanofi Aventis supported research projects by SL.

JP has been a consultant for and co-operated in clinical trials with AstraZeneca, Bristol Myers Squibb, Eli Lilly, Janssen-Cilag, Lundbeck, Pfizer, Sanofi Aventis. He has also received research grants from Astra Zeneca, Janssen-Cilag, Eli Lilly, Lundbeck and Sanofi-Aventis.

JT has served on the speaker bureau or advisory board for: Abbott, Addex Pharma, AGI Therapeutics, Almirall, Aryx, Astellas, Astra Zeneca, Bayer, Chugai, Danone, Ipsen, Menarini, Movetis, Norgine, Novartis, Nycomed, Ocera, Rose Pharma, SK Life Sciences, Smartpill, Sucampo, Theravance, Tranzyme, Xenoport, and Zeria.

CB, LD, KS, SVS, and MW have no conflict of interest related to this paper.

## Authors' contributors

MDH, LD and BP wrote the first and consecutive drafts of this paper. All other authors commented on this draft and contributed to the subsequent revisions. LD, CB and KS collected the data. MW performed the statistical analysis. SVS performed the radiological assessments. JT provided gastroenterological input. All other authors read and approved the final manuscript.

## Funding

The study was made possible with an unrestricted educational grant from Astra Zeneca.

## Pre-publication history

The pre-publication history for this paper can be accessed here:

http://www.biomedcentral.com/1471-230X/11/17/prepub
